# Enhanced Potency of a Broad H7N9-Neutralizing Antibody HNIgGA6 Through Structure-Based Design

**DOI:** 10.3389/fmicb.2020.01313

**Published:** 2020-06-19

**Authors:** Cong Chen, Zuliang Liu, Liguo Liu, Jianmin Wang, Qi Jin

**Affiliations:** ^1^NHC Key Laboratory of Systems Biology of Pathogens, Institute of Pathogen Biology, Chinese Academy of Medical Sciences and Peking Union Medical College, Beijing, China; ^2^Collaborative Innovation Center for Diagnosis and Treatment of Infectious Diseases, Hangzhou, China

**Keywords:** avian influenza virus, H7N9, human neutralizing antibody, structure-based design, enhanced potency

## Abstract

H7N9 influenza virus was first isolated in 2013 and has caused five epidemic waves among humans to date. Treatment opinions are currently limited. Previously, we characterized a human neutralizing antibody, HNIgGA6, by isolating rearranged heavy- and light-chain genes from convalescent patients. The mAb disrupts viral attachment to the cellular receptor by directly interposing into the receptor binding site (RBS) and broadly neutralizing divergent H7N9 strains. To increase the protective efficacy of HNIgGA6, we employed a structure-based design to enhance its binding affinity and neutralization potency. When the serine at position 28 on light-chain complementarity-determining region 1 (LCDR1) was substituted by a histidine, compared to HNIgGA6, the mutated antibody showed an approximately three-fold increase in HA-binding affinity and 10-fold enhancement in neutralization potency *in vitro*. Importantly, the S28H variant also exhibited broad H7N9-neutralizing activity. When administered to BALB/c mice, mAb S28H showed enhanced potency in inhibiting the pulmonary virus titre and reducing lung lesions and resulted in better protection of the animals than did the original antibody.

## Introduction

Avian influenza virus (AIV) uses birds or poultry as natural reservoirs and circulates among humans, causing acute respiratory infections. Sixteen HA subtypes (H1–H16) and nine NA subtypes (N1–N9) have been characterized. The identification of two influenza A virus genomes in bats, designated H17N10 (Tong et al., 2012) and H18N11 (Tong et al., 2013), demonstrated that bats might harbor more influenza virus genetic diversity than avian species. In March 2013, a reassortment H7N9 AIV subtype was first isolated in China and found to be highly contagious and lethal to humans (Liu et al., 2013; Rongbao et al., 2013). Since then, five major epidemic waves have been reported, resulting in a total of 1564 laboratory-confirmed human cases and at least 615 deaths^1^. A sudden increase in the number of human infections (688 cases) and a much broader distribution in the fifth wave was observed, causing concerns of a possible H7N9 pandemic (Quan et al., 2018). Due to the control of live poultry trade and poultry vaccinations, human cases of H7N9 infections have been significantly curtailed (Zeng et al., 2018; Wu et al., 2019). Only three cases in 2017-2018 and one case in 2019 were reported in China^2^. However, H7N9 virus has been evolving at a high rate. The virus showed improved ability to bind to the human α-2,6-linked sialic acid receptor and enhanced transmission to humans by carrying V186 and L226 in the receptor binding site (RBS) (Shi et al., 2013). High-pathogenicity (HP)-H7N9 variants, which possessed an insertion of several additional basic amino acid residues at the hemagglutinin (HA) cleavage site, emerged in wave 5 and were highly pathogenic for chickens (Yang et al., 2017; Qi et al., 2018). More importantly, some HP-H7N9 isolates were able to be transmitted among ferrets via respiratory droplets and were more pathogenic to mammals (Imai et al., 2017). H7N9 has also acquired some other mammalian-adapted genetic changes, including T271A, K526R, A588V, E627K, or D701N in the viral PB2, which facilitated viral replication and increased viral-induced disease severity in mammals (Mok et al., 2014; Song et al., 2014; Bi et al., 2015; Quan et al., 2018). Neuraminidase inhibitor (NAI) resistance (R292K substitution in NA) was detected in both NA inhibitor-treated patients (Hu et al., 2013; Yen et al., 2013) and a few nonhuman-derived HP-H7N9 strains (Quan et al., 2018). Varied virulence and transmissibility has been confirmed in animal models by using circulating H7N9 viruses from wave five (Bao et al., 2019; Sun et al., 2019). Therefore, H7N9 AIV is classified as “an unusually dangerous virus for humans” by the World Health Organization (WHO,^3^).

Ribavirin was once used to treat H7N9 infection; however, this polymerase inhibitor merely showed effectiveness in mild cases (Yu et al., 2013). NAI (e.g., oseltamivir and zanamivir) are currently the primary therapeutic agents used in the clinic against H7N9 infection (Hu et al., 2013). Multiple strategies to develop vaccines against H7N9 influenza have been conducted to date (Bart et al., 2014; Chen et al., 2014; Leung et al., 2015; Yang et al., 2018). A series of HA-directed neutralizing antibodies (nAbs) that exert antiviral activity *in vitro* and *in vivo*, including m826 (Yu et al., 2017), HNIgGD5 (Wang et al., 2015), H7.167 (Thornburg et al., 2016), 1A8 (Stadlbauer et al., 2018), etc., have also been characterized. These nAbs are ideal for prophylaxis, especially for those individuals at risk of postexposure, or for an alternative or adjunct to H7N9 interventions.

Our lab characterized a HA-targeted monoclonal antibody (mAb), HNIgGA6, by isolating rearranged genes for heavy and light immunoglobulin chains recovered from H7N9 (A/Anhui/1/2013, H7N9-AH) infected patients in the convalescent phase (Chen et al., 2015). The mAb broadly neutralized divergent H7N9 strains from 2013 to 2017 (Chen et al., 2018b). The crystal structure of the HNIgGA6/HA1 complex (PDB ID: 5XKU) indicated that the mAb recognized viral HA by interposing its heavy-chain complementarity-determining region three (HCDR3) directly into the RBS and thus blocking cell attachment (Chen et al., 2018a). Based on the structure of the HNIgGA6/HA1 complex, we attempted to obtain a more potent neutralizing monoclonal antibody. Several models were proposed in this study. When the serine was substituted by histidine at position 28 on the light-chain complementarity-determining region 1 (LCDR1), the S28H variant presented ∼10-fold higher neutralizing activity and retained the neutralizing breadth of the parent HNIgGA6. More importantly, the S28H variant also exhibited enhanced potency in inhibiting the pulmonary virus titre and reducing lung lesions and resulted in better protection of the animals when administered to BALB/c mice. Our findings show that a structural biology-based optimization can be used to improve the manufacturing characteristics of an antibody and to provide a better understanding of RBS-targeted antibodies and the mechanism of neutralization by the HNIgGA6 antibody.

## Results

### Structure-Based Optimization of HNIgGA6

**FIGURE 1:**
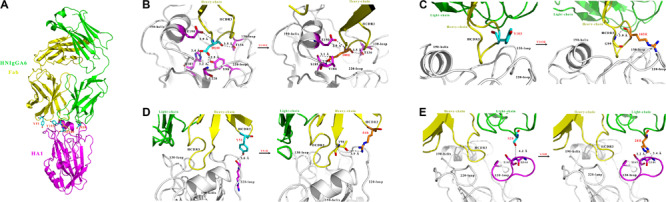
Structure-based design of HNIgGA6. **(A)** Crystal structure of the globular head HA1 of H7N9-AH with HNIgGA6 Fab complex (PDB ID: 5XKU). The head is represented in magenta, the heavy-chain is yellow and the light-chain is green. **(B–E)** Structural basis for residues D100, Y103, Y51 and S28 in antibody recognition (left). The heavy-chain (yellow), light-chain (green) and HA1 (magenta and gray) are shown in cartoon. The 130-loop, 190-helix and 220-loop are labeled. The interacted residues on HA are shown as sticks in magenta. The proposed modes for 100E, 103R, 51R, and 28H in binding with HA1 are displayed (right). Residues D100, Y103, Y51, and S28 are shown as sticks in cyan, and their substitutions (100E, 103R, 51R, and 28H) are shown as sticks in orange. Potential hydrogen bonds are represented by dashed lines and marked with distances. The mutated residues are produced by PYMOL program.

We previously characterized the structure of HNIgGA6 complexed with HA1 and showed that HNIgGA6 recognizes viral RBS mostly through its HCDR3 (Figure 1A). From the structure of the complex, we identified that residue D100 utilizes a network of hydrogen bonding interactions in viral RBS with four residues (Y98, H183, V186, and G228). To increase the binding affinity of HNIgGA6 to HA1, we mutated D100 to 100E. The side chain of glutamic acid could be interposed deeper into the hydrophobic pocket than that of aspartic acid and strengthen hydrogen bonding (Figure 1B). No direct contact with viral RBS was observed for Y103. However, when the residue was substituted with arginine, the viral RBS could bind to residue G99 in HCDR3 via hydrogen bonds and thus stabilize the antibody conformation (Figure 1C). Similarly, another residue at Y51 in HCDR2 was also mutated to arginine. The hydrophobic surface of 51R could enhance the interaction of HCDR2 with HCDR3 and help the antibody loop to enter the receptor binding groove on HA1 (Figure 1D). The antibody light chain is not as involved in binding with the RBS as extensively as the heavy chain. To enhance its interaction with the viral antigen, we mutated S28 on LCDR1 to a histidine, which could create a hydrophobic environment for contact with viral HA1 (Figure 1E).

### Enhancement of Viral HA Binding Affinity Through the Alteration of Ser28 on LCDR1

**FIGURE 2:**
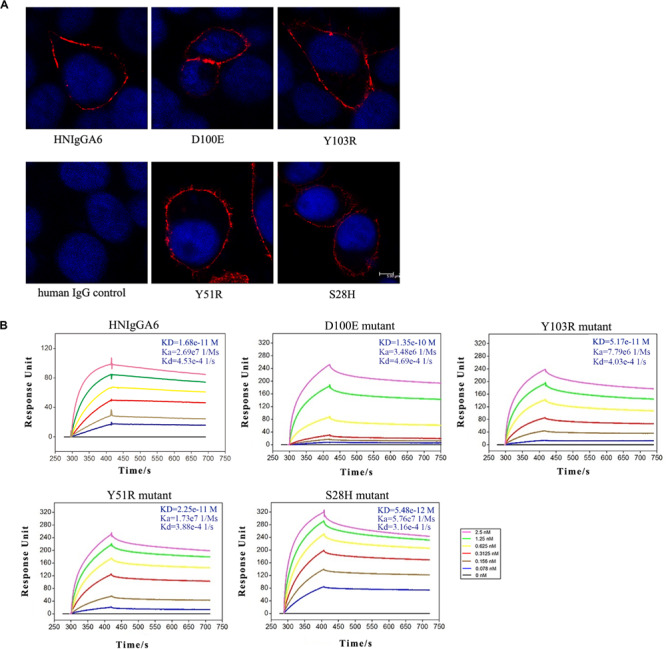
Enhancement of viral HA binding affinity for the S28H mutant. **(A)** Viral HA proteins were expressed in HeLa cells and detected using IFA by HNIgGA6 and the four variants as indicated. **(B)** Binding of HNIgGA6 and four variants to HA1 of H7N9-AH was measured by using surface plasmon resonance measurements with BIAcore 3000 analysis software. The KD value was calculated with a simultaneous kinetic Kd (dissociation rate; Koff)/Ka (association rate; Kon) model.

Based on the crystal structure of the HNIgGA6/HA1 complex, mutations at Y51R, D100E and Y103R in HCDRs and at S28H in LCDR1 were constructed. The four mutated antibodies were purified, and their HA binding capabilities were checked by IFA. As shown in Figure 2A, all four variants recognized viral HA, similar to the parent HNIgGA6. Their binding affinity to viral HA1 was further detected by SPR experiments (Figure 2B). We found that the D100E mutant on HCDR3 had a much lower binding capacity to that of the wild-type HNIgGA6. A slight increase in binding affinity was observed with Y103R on HCDR3 and Y51R on HCDR2. Strikingly, when serine was substituted by histidine at position 28 in LCDR1, the mutated antibody exhibited a noticeable increase in binding affinity (*K*_*D*_ is 5.48e-12 M), which was an approximately three-fold increase compared to HNIgGA6.

### Enhancement of the Neutralizing Potency for the S28H Variant

**FIGURE 3:**
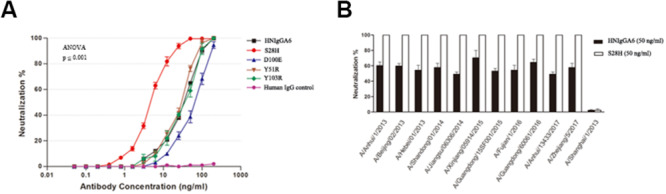
Enhancement of *in vitro* neutralizing potency for the S28H variant. **(A)** Neutralizing activities of four HNIgGA6-variants against H7N9 pseudovirus were tested on MDCK cells. An irrelevant human IgG was used as a negative control. One-way ANOVA was used to analyze the data (ANOVA, *F* = 2448.8, *p* = 1.29E-17). **(B)** S28H neutralized 11 out of the total 12 strains tested.

The anti-H7N9 neutralizing activity for the mutated antibodies was also assessed with MDCK cells. All four mutants were able to neutralize the H7N9-AH pseudovirus in a dose-dependent manner similar to wild-type HNIgGA6, and the S28H mutant had the most potent neutralizing activity. The IC_50_ value for the S28H mutant was 4.38 ng/ml, compared to 41.66 ng/ml for HNIgGA6, indicating that S28H has a 10-fold more potent neutralization potency *in vitro* (Figure 3A). The neutralizing activity of S28H against other H7N9 strains was also tested. Total 12 H7N9 pseudoviruses, each carrying distinct mutations in viral HA, was generated as previously described (Chen et al., 2018b) (Supplementary Figure S1). As shown in Figure 3B, similar to its parent HNIgGA6, S28H neutralized most of the H7N9 strains from 2013 to 2017.

### Improvement of the *in vivo* Neutralization Potency of the S28H Variant

**FIGURE 4:**
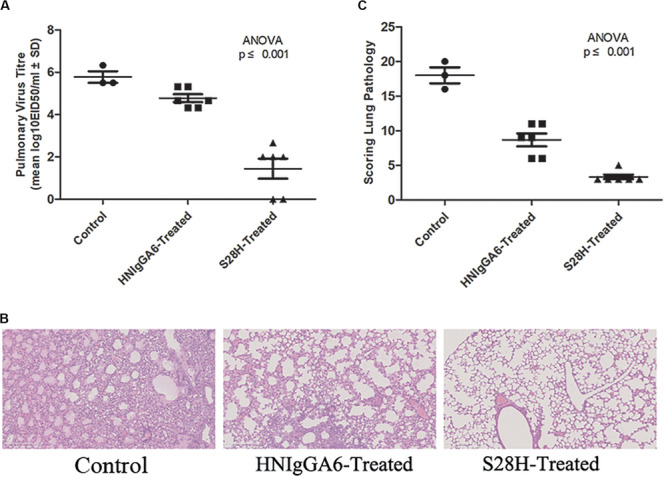
Increased *in vivo* neutralization potency of the S28H variant. Mice were passively immunized with HNIgGA6 or S28H variant 24 h and then challenged with a lethal dose of H7N9 virus. **(A)** Pulmonary virus titres were determined. Virus titres were substantially reduced in the S28H-treated group compared to HNIgGA6-treated and the control mice. One-way ANOVA was used to analyze the data (ANOVA, *F* = 37.33, *p* = 7.05E-06). **(B)** Pathological changes in the lungs of the mAb-treated mice compared with the control group were detected. The represented results were shown. **(C)** Histopathological changes in the lungs were scored. Less dramatic pathological changes in the lungs of the S28H-treated mice were detected One-way ANOVA was used to analyze the data (ANOVA, *F* = 70.52, *p* = 2.32E-07).

To determine the *in vivo* neutralization potency of the S28H variant, six mice were passively immunized with HNIgGA6 or S28H mAb by intraperitoneal injection at a final concentration of 5 mg kg^–1^. Additionally, the control group was treated with an equal volume of PBS. The mice were then infected with 2 × LD_50_ of H7N9 virus at 24 h later. All animals were necropsied at 5 days post infection (d.p.i.) and the lungs were removed to determine the pulmonary virus titres. In the HNIgGA6-treated and the control group, high pulmonary virus titres were detected, while three control mice died from viral infection (Figure 4A). In contrast, viral proliferation was substantially inhibited by the S28H mAb and the viral titres were reduced by more than three orders of magnitude (Figure 4A). Severe lung tissue damage was also observed in association with high viral load in the control animals. As shown in Figure 4B, H7N9 infection resulted in dramatic bronchial epithelial cell necrosis, diffuse alveolar septum widening, alveolar septum and peribronchial inflammatory cell infiltration of the control mice. Partial bronchial epithelial cell necrosis, local alveolar septum widening, alveolar septum and inflammatory cell infiltration were also observed in the HNIgGA6-treated mice. In contrast, although local alveolar septa can be seen with mild widening, the overall lesion was significantly inhibited in the mice that were immunized with the S28H mAb. These observations were further verified by scores of the entire histopathological change. Passive immunization with either HNIgGA6 or S28H variant had lower pathology scores compared with the control group, while S28H showed stronger inhibition of lung lesions due to more potent H7N9-neutralizing activity (Figure 4C).

### Protection of H7N9-Infected Mice by Optimized S28H mAb

**FIGURE 5:**
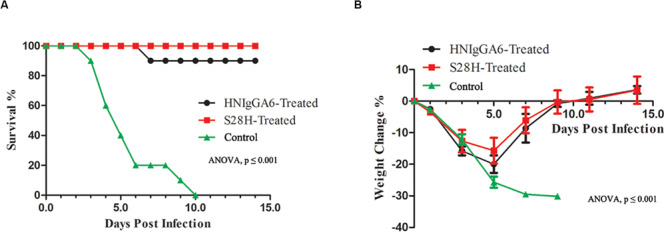
The S28H mAb protected mice from H7N9 infection. Mice received HNIgGA6 or S28H mAb 24 h before H7N9 infection and were monitored daily for 14 days. **(A)** The survival rate is presented. One-way ANOVA was used to analyze the data (ANOVA, *F* = 20.26, *p* = 1.02E-06). **(B)** The average body weight change of the mice is shown. One-way ANOVA was used to analyze the data (ANOVA, *F* = 14.52, *p* = 3.87E-11).

Finally, the protective efficacy of the S28H mAb was tested. Fifteen mice were passively immunized with the mAbs at 5 mg kg^–1^ one day before the H7N9 challenge. Mouse body weight change and the survival rate were monitored. As expected, both HNIgGA6 and its S28H variant conferred substantial protection of the mice. In the control group, death appeared as soon as two dpi until all the animals had succumbed to H7N9 infection by 10 dpi (Figure 5A) and mouse body weight declined rapidly (Figure 5B). Except for one mouse in the HNIgGA6-treated group that died during the test, all other mice survived H7N9 infections (Figure 5A). In the first few days, a pronounced weight loss was also observed in the antibody-treated groups; however, the mice gradually restored their body weights, suggesting the recovery of the infected animals (Figure 5B). Together, these results demonstrated that HNIgGA6 and its S28H mutant substantially decreased the mortality and morbidity of mice infected with H7N9.

## Discussion

Strategies to combat the avian A H7N9 influenza and to prevent further spread are currently needed. Several studies with animal models have shown that nAbs can prevent H7N9 infections, reduce lung lesions, and protect animals (Wang et al., 2015; Thornburg et al., 2016; Yu et al., 2017; Amanat et al., 2019). We previously characterized a human H7N9-neutralizing antibody, HNIgGA6, which broadly neutralized divergent H7N9 strains from the five epidemics (Chen et al., 2015, 2018b). In the present study, we employed a structure-guided design to enhance the neutralization potency and to increase the protective efficacy of HNIgGA6.

The mAb HNIgGA6 was able to achieve high-affinity recognition of viral HA protein and the neutralization of the H7N9 virus mostly by its HCDR3 (Chen et al., 2018a). The level of receptor mimicry has spatial limitations, as there is only space for a single antibody loop to enter the binding groove. Based on the structure analysis, four mutations were explored to improve its binding affinity with viral HA. In HCDR3, both D100E and Y103R mutants retained the capacity to bind to mAb HNIgGA6, suggesting that it might be difficult to alter the amino acid identity in HCDR3 to achieve greater affinity (Figure 2B). Although there is only space for a single antibody loop to enter the RBS, it may not be possible to ignore the effect of other CDRs in both the heavy and light chains. When tyrosine was mutated to arginine at position 51 in HCDR2, it bound viral HA with an affinity of 2.25e-11 M, similar to that of the wild-type HNIgGA6. Strikingly, when serine was mutated to histidine at position 28 on LCDR1, the antibody exhibited an ∼three-fold higher HA-binding affinity and a 10-fold increase in virus neutralizing activity compared to the wild-type (Figures 2B, 3A). The S28H mutation created a hydrophobic environment for residues G144 and S145 of HA and stabilized the 130-loop conformation, which might affect its subsequent affinity to HNIgGA6. More importantly, the amino acid substitution at S28H improved potency without disturbing the neutralization breath of HNIgGA6. The variant neutralized divergent H7N9 strains from 2013 to 2017 (Figure 3B). The only isolate showing resistance was A/Shanghai/1/2013 (H7N9-SH1). H7N9-SH1 carries 186G and 226Q in viral RBS, which resulted in the production of escape mutant (Chen et al., 2015, 2018b). Analysis of antibodies from mutation sites suggests an important role for the light-chain in achieving high-affinity binding and enhanced potency. These results provide a deeper insight into RBS-targeted antibodies and may also be used as a guide for the rational design of therapeutic molecules.

To investigate *in vivo* neutralization efficacy, the S28H variant was further tested by using BALB/c mice models. As seen in Figure 4, with the S28H mutation on the light chain, the variant showed enhanced potency in inhibiting pulmonary virus proliferation and lung lesions compared to the parent HNIgGA6. While all mice in the control group succumbed to H7N9 infection within 10 days, the mice successfully survived until the end of the test due to the protection of the mAbs (Figure 5). Although, the differences in survival rate were slightly between S28H- and HNIgGA6-treated mice, these results together demonstrated enhanced *in vivo* potency of the optimized S28H mutant.

Low-pathogenicity (LP)-H7N9 AIVs are still dominant in China. Human infections with HP-H7N9 have also been reported in several provinces of Mainland China^4^. Although there was no substantial evidence showing that HP-H7N9 was more virulent for humans, the emergence of HP-H7N9 results in a new threat to both agriculture and public health. Viral NA and other internal genes of H7N9 have also undergone a dynamic reassortment (Wang et al., 2014; Zhu et al., 2014; Quan et al., 2018). The mAb HNIgGA6, which is capable of neutralizing both LP- and HP-H7N9, might serve to prevent or treat H7N9 infection (Chen et al., 2018b). A structure-guided design has been utilized to optimize the solubility and to improve the neutralization potency of HIV-neutralizing antibodies (Rudicell et al., 2014; Kwon et al., 2016). In this study, based on the structure of HNIgGA6/HA1, we took advantage of a structure-guided approach for enhancing neutralization potency. The S28H variant was generated, and successfully increased the *in vitro* HA-binding affinity and correlated with substantially increased neutralization potency *in vivo*.

HNIgGA6 and its derivative S28H mutant were originally isolated from H7N9-infected patients and broadly neutralized divergent H7N9 strains from 2013 to 2017. Considering that there is still a lack of licensed H7N9 vaccines, H7N9-neutralizing antibodies may be able to protect human from viral infection. Passive immunization could be especially helpful for individuals at high risk of postexposure or could be used together with antiviral drugs to prevent H7N9-infection. Notably, this study provides a better understanding of the mechanism of protective antibody recognition and a sound foundation for the design of therapeutic drugs and vaccines.

## Materials and Methods

### Ethics Statement

This study was approved by and executed under the supervision of the Ethics Committee of the Institute of Pathogen Biology at the Chinese Academy of Medical Sciences and Peking Union Medical College. The use of murine animals was approved by the Institutional Animal Care and Use Committee of the Institute of Laboratory Animal Science at Peking Union Medical College. Female 6-week-old specific pathogen-free BALB/c mice (Institute of Laboratory Animal Sciences, Beijing, China) were used in this study. All experiments were performed in an animal biosafety level three facility and in accordance with the Chinese national guidelines for the care of laboratory animals, as described previously (Chen et al., 2015).

### Cells and Viruses

MDCK and HEK293T (293T; American Type Culture Collection) cells were maintained in Dulbecco’s modified Eagle’s medium (DMEM; HyClone) supplemented with 10% fetal bovine serum (FBS; Invitrogen), 100 U ml^–1^ penicillin and 100 mg ml^–1^ streptomycin at 37°C with 5% CO_2_. The H7N9 strain A/Anhui/1/2013 was used in this study.

### Protein Expression and Purification

The globular head HA1 of H7N9-AH (residues 47 to 322, based on H3 numbering) was amplified by PCR and inserted into the pFastBacI vector (Invitrogen). An N-terminal gp67 signal peptide and a C-terminal His6x tag were anchored to the HA1 gene. The recombinant bacmid was transfected into Sf21 insect cells, and the protein was purified by nickel-nitrilotriacetic acid (Ni-NTA) affinity chromatography followed by size exclusion chromatography on a Superdex 200 column (GE Health Care). The heavy- and light-chain genes for the mAb HNIgGA6 and the four variants were cloned into the antibody expression vector and expressed by transient transfection in HEK293F cells by using Lipofectamine 2000 (Invitrogen). The cell culture was collected at 72 h later and centrifuged to remove cell debris. Human IgG1 protein was then purified by affinity chromatography using Protein A agarose (TransGen Biotech) and further purified by size exclusion chromatography. Protein concentration was measured spectrophotometrically (GE Healthcare).

### Immunofluorescence Detection

The full-length viral HA gene was cloned into the pCDNA3.1 vector and transfected into HeLa monolayer cells by using Lipofectamine 2000 (Invitrogen). At 24 h post transfection, the cells were fixed with 4% paraformaldehyde and washed with PBS for three times. The slides were then incubated with HNIgGA6 or the mutated antibodies at 37°C for 1 h. Bound antibodies were detected with rhodamine-conjugated goat anti-human IgG antibody (Invitrogen) and observed under a fluorescence microscope. The represented results are shown.

### Surface Plasmon Resonance Binding

The purified mAbs were first immobilized on a CM5 chip (GE Healthcare). Viral HA1 protein was exchanged in PBST buffer (phosphate-buffered saline with 0.005% (v/v) Tween-20, pH 7.4) by gel filtration before use. To test the binding affinity of the mAbs to HA1, the HA1 protein was serially diluted to a series of concentrations between 0.078 and 2.5 nM (0.078, 0.156, 0.3125, 0.625, 1.25, and 2.5 nM) and then flowed over the mAb. The binding kinetic was analyzed with BIAcore 3000 analysis software (BIAevaluation Version 4.1), by using a 1:1 Langmuir binding model.

### Pseudovirus-Based Neutralization Assay

To generate pseudovirus for H7N9-AH, viral HA and NA genes were cloned into the pcDNA 3.1 expression vector and verified for sequences. The HA- and NA-expressing vectors were then cotransfected with a pNL4-3-Luc-R-E- viral backbone plasmid into 293T cells using Lipofectamine 2000 (Invitrogen) for 48 h. H7N9 pseudoviruses in the supernatants were collected and viral infection was quantified by the luciferase activity in MDCK cells. The viral titre was determined as the median tissue culture infective dose (TCID_50_). The pseudoviruses for H7N9 strains were generated as previously described (Chen et al., 2018b). Briefly, HAs of H7N9 strains (A/Shanghai/1/2013, A/Beijing/02/2013, A/Hebei/01/2013, A/Shanghai/01/2014, A/Jiangsu/06306/2014, A/Xinjiang/05914/2015, A/Guangdong/15SF001/2015, A/Fujian/1/2016, A/Guangdong/6006/2016, A/Anhui/13433/2017, and A/Zhejiang/5/2017) were constructed by site-directed mutagenesis of the HA-encoding gene of the H7N9-AH. H7N9 pseudoviruses were then constructed by using different HAs and NA of H7N9-AH. In a pseudovirus-based neutralization assay, 100 × TCID_50_ of pseudovirus was incubated with serially diluted antibodies at 37°C for 1 h. An irrelevant human IgG protein was used as a negative control. The mixture was then added to the culture of MDCK cells. At 48 h post infection, luciferase activity was quantified to determine the neutralization potency.

### Viral Infection and Antibody Treatment of Mice

For each group, 15 mice were used and raised in three independent groups. The animals were intraperitoneally injected with HNIgGA6 or the S28H variant at a concentration of 5 mg kg^–1^, and the control group was treated with PBS which was used to dissolve the antibodies. Twenty-four hours later, all animals were challenged intranasally with 50 μl of a 2 × LD_50_ mouse infectious dose of H7N9 virus under anesthesia with isoflurane. Signs of disease and mortality were monitored every day for up to 14 days, while mouse body weight was weighed every 2 days to avoid overdisturbing.

### Virus Titres and Histopathology Detection

Animals were anesthetized and necropsied at 5 days post infection. To determine pulmonary virus titres, lungs were removed into PBS and homogenized. Ten-fold serial dilutions of the virus were used to inoculate SPF chicken embryos at 37°C for 72 h. Virus titres were then determined by 50% egg infective dose (EID_50_) according to the Reed and Muench method. To determine the histology in the lungs, the tissues were fixed in 10% neutral-buffered formaldehyde and subsequently paraffin embedded. The sections (5 μm) were stained with hematoxylin and eosin. Histopathological change was analyzed microscopically and scored on a scale of 0 (no change) to four (maximum inflammation) according to previously described methods (Chen et al., 2015).

### Statistical Analysis

The virus titre and lung pathology score were measured independently for each of the infected mice. Statistical analysis was calculated by using Statistical Package for the Social Sciences software. Differences with *P* < 0.05 (ANOVA) were considered statistically significant.

## Data Availability Statement

All datasets presented in this study are included in the article/Supplementary Material.

## Ethics Statement

The animal study was reviewed and approved by the Institutional Animal Care and Use Committee of the Institute of Laboratory Animal Science at Peking Union Medical College.

## Author Contributions

QJ and JW conceived, designed the study. CC, ZL, and LL performed the experiments. All authors read and approved the manuscript.

## Conflict of Interest

The authors declare that the research was conducted in the absence of any commercial or financial relationships that could be construed as a potential conflict of interest.

## Footnotes

1 http://www.who.int/csr/don/05-september-2018-ah7n9-china/en/

2 https://www.who.int/influenza/human_animal_interface/Influenza_Summary_IRA_HA_interface_09_04_2019.pdf?ua=1

3 http://www.sciencemag.org/news/2013/04/h7n9-unusually-dangerous-virus-international-group-experts-concludes

4 http://www.who.int/influenza/human_animal_interface/HAI_Risk_Assess ment/en/

## Supplementary Material

FIGURE S1 Phylogenetic analysis of the HA genes of representative H7N9 viruses collected from 2013 to 2017.
