# Supplementary figures and images for: Enhanced Potency of a Broad H7N9-Neutralizing Antibody HNIgGA6 Through Structure-Based Design
Source: Front Microbiol. 2020 Jun 19;11:1313. doi: 10.3389/fmicb.2020.01313 (PMC7316883; doi:10.3389/fmicb.2020.01313)

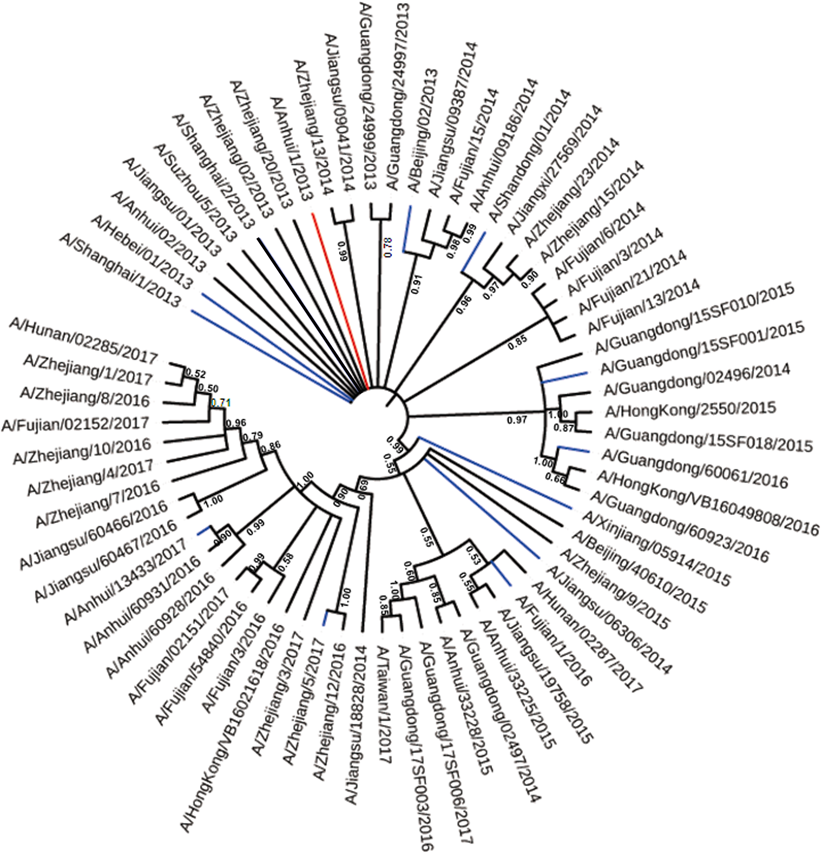

Supplement: FIGURE S1 — Phylogenetic analysis of the HA genes of representative H7N9 viruses collected from 2013 to 2017. [file Image_1.tif]
